# Simultaneous treatment of two lung cancer lesions with stereotactic MR-guided adaptive radiation therapy: A case report

**DOI:** 10.1097/MD.0000000000032626

**Published:** 2023-01-13

**Authors:** Frank Chen, Yuan-Kai Cheng, Chen-Han Chiang, Tzu-Ying Lu, Chih-Jen Huang

**Affiliations:** a Department of Radiation Oncology, Kaohsiung Medical University Hospital, Kaohsiung Medical University, Kaohsiung, Taiwan.

**Keywords:** lung cancer, MR-linac, radiotherapy, SABR, simultaneously

## Abstract

Lung cancer is 1 of the most prevalent cancers globally. Definitive stereotactic ablative radiotherapy (SABR) is suggested for those who are unfit for or refuse surgical intervention. Here we present a patient with 2 lung cancer lesions who received SABR simultaneously with magnetic resonance Linear accelerator (Linac)-magnetic resonance (MR).

**Patient concerns:**

A 46-years-old man had history of left lower lung cancer post lobectomy in 2018. Two recurrent tumors were found 2 years following, then became enlarged 4 months later.

**Diagnoses:**

The recurrent tumors were found by computed tomography.

**Interventions:**

SABR was indicated due to inoperability and small size. Simulation was done both by computed tomography and MR scan with ViewRay MRIdian Linac, with the prescription dose being 50 gray in 4 fractions performed every other day within 2 weeks. The 2 lesions were irradiated at the same time with a single isocenter with mean treatment time was 78 minutes.

**Outcomes:**

No acute side effect was noted. Follow-up chest computed tomography scan 14 months after SABR showed mild consolidation and pneumonitis over the upper irradiated site favoring radiation-related reasons, while pneumonitis was resolved over the lower irradiated site. Positron emission tomography showed no definite evidence of FDG-avid recurrence. The patient has survived over 18 months following SABR and more than 4 years from the first diagnosis of lung cancer without significant adverse effects.

**Lessons:**

Simultaneous SABR for multiple lung lesions is quite challenging because tumor motion by breathing can increase the risk of missing the target. With help by MR-Linac, simultaneous SABR to multiple lung lesions can be performed safely with efficacy.

## 1. Introduction

Lung cancer is 1 of the most prevalent cancers worldwide, and has been 1 of the leading causes of cancer-related deaths in men and women.^[1]^ Recently, due to more prevalence of low-dose computed tomography (CT), more non-small-cell lung cancers (NSCLC) have been diagnosed. Surgery is the main treatment for operable tumors, and definitive radiotherapy (RT) is suggested for those unfit for or refusing surgical intervention.

Among all RT techniques, stereotactic ablative radiotherapy (SABR), also called stereotactic body radiotherapy is preferred than other techniques in treating lung lesions, since SABR could cause good local control^[2]^ and better survival and lower rates of toxicity than conventional fractionated radiotherapy.^[3]^ A recent prospective observational trial showed 5-year overall survival of SRBT was 87%, which was comparable to that of the propensity-matched VATS cohort (84%).^[4]^ SABR uses higher dose per fraction with less fractions needed, thereby avoiding further effective DNA damage regardless of cell cycle and oxygen status, and making treatment more convenient for the patient. Due to the higher dosage per fraction, accuracy and high dosage rate of the equipment are critical. Many modalities are being used for SABR, as for example, the linear accelerator (Linac), which has high dose rate delivery, imaging options and gating or tracking mechanisms, can be used for SABR.

SABR for lung lesions is quite challenging because respiratory motions can increase the risk of missing the target, and thus a larger radiation field may be needed, which might also increase the risk of radiation pneumonitis and other adverse events.^[5]^ As a result, proper modality should be used for lung SABR. Newly developed magnetic resonance (MR)-Linac has certain characteristics, and there have been some publications showed that MR-Linac is a ideal modality for lung SABR. Here we present a patient with 2 lung cancer lesions who received SABR simultaneously with MR-Linac. Ethical approval to report this case was obtained from Kaohsiung medical university hospital (KMUHIRB-E(I)-20220102). Written informed consent was obtained from the patient for his anonymized information to be published in this article.

## 2. Case presentation

A lung mass in the left lower lobe was incidentally found in a 46-years-old man without any underlying diseases on undergoing a health examination in 2018. He received a lobectomy by video-assisted thoracoscopic surgery, and pathological analysis showed adenocarcinoma, pT1cN0M0, stage 1A. During a regular follow-up at 2 years, 2 recurrent tumors were found over the left upper lobe (long axis 0.6 cm, 0.6 cm), and these tumors enlarged 4 months later (long axis 0.95 cm, 0.65 cm). Due to tumor size enlargement, malignancy was highly suspected. The chest surgeon transferred the patient to the Radiation Oncology department because the tumors were difficult to resect surgically and had a high possibility of tissue adhesion caused by the previous operation. Since these tumors were quite small, SABR to these tumors was indicated.

**Figure 1.**
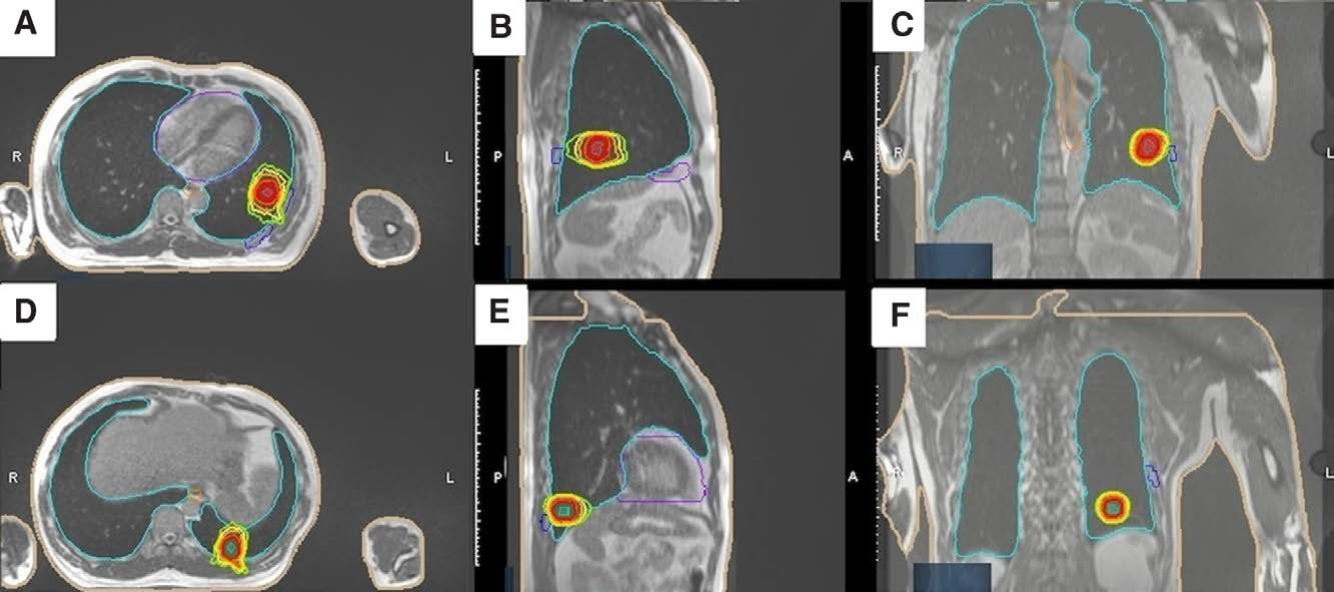
Isodose curves of treatment plan for the two lesions. Transverse (A), sagittal (B), and coronal (C) view of upper lesion. Transverse (D), sagittal (E), and coronal (F) view of lower lesion.

**Figure 2.**
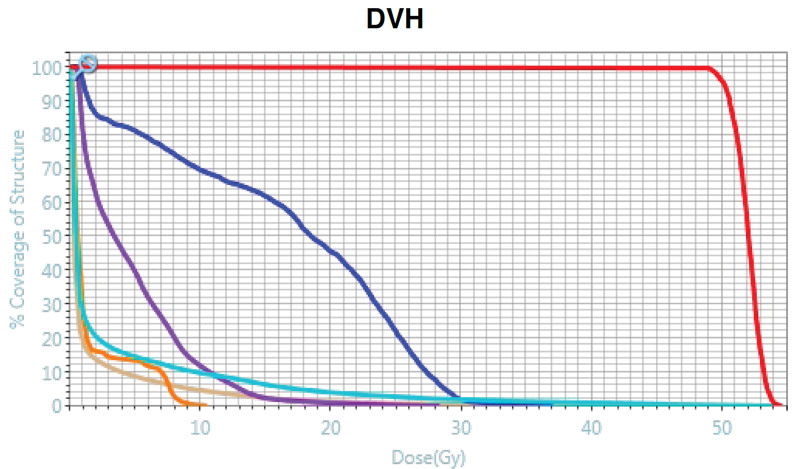
Dose-volume histogram of treatment plan. Red line: PTV; dark blue line: rib; purple line: heart; light blue line: whole lung; orange line: esophagus; brown line: skin. PTV = planning target volume.

Simulation was done both by CT and MR scan. The patient’s geometry was in the supine position with left arm up and deep-inspiration breath-holding. CT simulation was performed with thickness of 2 mm; then, MR simulation was done with 0.35 *T* magnetic field, and true fast imaging with steady precession sequence (True FISP) MR scan (25 seconds) was acquired on the ViewRay MRIdian® Linac (Oakwood Village, OH), with deep-inspiration breath-holding and thickness of 1.5 mm. No immobilization device was used since magnetic resonance imaging (MRI) was performed every fraction for inter and -intrafraction motion management respectively. All contours were delineated on the True FISP MR simulation scan, and the simulation CT was exported to MRIdian treatment planning system and deformably registered to the MR simulation scan for electron density information for dose calculation purposes. Gross target volume (GTV) was defined as gross tumors seen on simulation CT or MR scans, which was uniformly expanded by 3 to 5 mm to create the planning target volume (PTV). The PTV volume for 2 lesions were 6.6 mL and 5.0 mL. Organs at risk OAR (OAR) including normal lung, heart, spinal cord and rib were also carefully contoured. Prescription dose for PTV was 50 gray (Gy) in 4 fractions (biologically effective dose = 112.5 Gy, equivalent dose in 2 Gy fractions = 93.75, with *α*/*β* = 10 Gy.), and the treatment was performed every other day within 2 weeks. The MRIdian planning system was used to generate an intensity modulated radiation step-and-shoot treatment plan based on a Monte Carlo dose calculation with magnetic field corrections (shown in Fig. 1). The mean dose, V10 Gy and V20 Gy of whole lung were 2.99 Gy, 9.72% and 3.94% (shown in Fig. 2).

For every treatment, a breath-hold MR scan was acquired to define the anatomy-of-the-day prior to each fraction. The GTV was rigidly co-registered to the baseline MRI, followed by an online couch shift. OAR contours were deformably registered to the daily MR scan. We manually edited the GTV, PTV every fraction if needed, and the average volumes of recontoured PTV for 2 lesions were 6.7 mL (6.6–6.9 mL) and 5.1 mL (5–5.3 mL) respectively. Deformed OAR contours in the proximity of the PTV were also edited. Next, treatment plans were reoptimized using the MRIdian planning software, with the same beam parameters and optimization objectives as in the baseline plan.

During beam on time, real-time tissue tracking on sagittal image acquisition every 250 ms was performed to automatically gate the treatment delivery. The tracking region of interest was defined daily from the GTV. Beam delivery was automatically paused when > 5% of the tracking region of interest was out of PTV with deep-inspiration breath-holding. Since the real-time image was only provided with the sagittal view, we could not determine a proper image slice for us to track 2 lesions concurrently, so during simulation, we use sagittal cine MR image of MRIdian to observe the tracks of 2 lesions while free breathing was performed. We found the 2 lesions moved consistently under free breathing with 3 cm along the cranial-caudal axis, which allowed us to track the larger lesion only with confidence during treatment. The 2 lesions were irradiated at the same time, and the mean treatment duration for 4 fractions was 78 minutes. No acute side-effects were noted during and after SABR.

**Figure 3.**
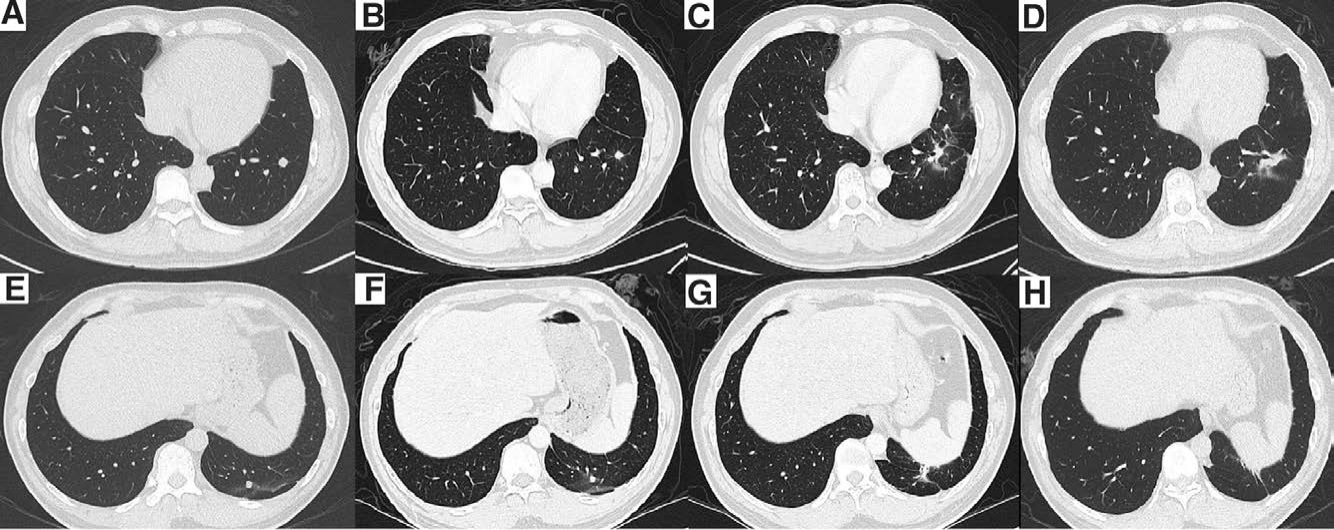
Serial images of chest CT. CT scans of upper lesion at pre-SABR (A), one month after SABR (B), seven months after SABR (C) and fourteen months after SABR (D). CT scans of lower lesion at pre-SABR (E), one month after SABR (F), seven months after SABR (G) and fourteen months after SABR (H). CT = computed tomography, SABR = stereotactic ablative radiotherapy.

**Figure 4.**
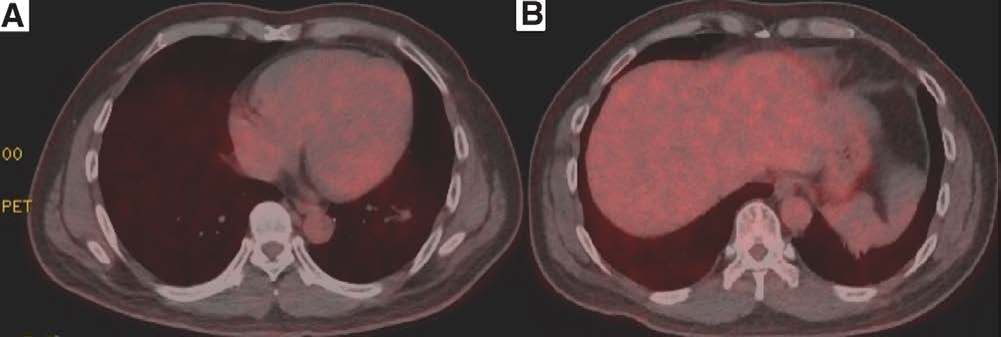
Post-SABR PET images for upper lesion (A) and lower lesion (B). PET = positron emission tomography, SABR = stereotactic ablative radiotherapy.

The patient received 4 courses of gemcitabine and cisplatin/carboplatin thereafter. Follow-up chest CT scan done 1 month after completion of SABR showed no obvious changes. Seven months later, CT scan showed mild pneumonitis and consolidation near the 2 lesions. CT scan 14 months after SABR showed persistent consolidation and shrinkage over the upper irradiated site with pneumonitis, appearing radiation-related, although the pneumonitis was resolved over the lower irradiated site (shown in Fig. 3). To see whether the lesions were viable, positron emission tomography (PET) was arranged, which showed no definite evidence of recurrence (shown in Fig. 4). After SABR, the patient’s daily activity was not influenced and he was still able to do his original job. He received regular follow-up at our Radiation Oncology department. He has survived over 18 months following SABR and more than 4 years from the first diagnosis of lung cancer without significant adverse effects.

## 3. Discussion

SABR has been recommended and widely used for inoperable early-stage NSCLC for its non-invasiveness and effectiveness. Many modalities can be used for lung SABR. Linacs, which can deliver highly conformal plans with high dose rates with the combinations of various imaging options (such as cone beam CT, fluoroscopy, and MV/kV imaging) as well as gating or tracking mechanisms (such as external fiducial-or surface-based optical tracking and internal fiducial-based radiofrequency tracking), have been used for lung SABR. For decreasing tumor motion, breath-holding techniques such as active breathing control, compression techniques, audio-visual coaching with feedback, and automatic gating have long been utilized.^[6]^ Breath-hold gated delivery can reduce respiratory motion for minimizing PTV, and has proven dosimetric benefits.^[7]^ PTV significantly correlates with the incidence of radiation pneumonitis,^[5]^ especially for those with multiple lung tumors, reduced lung capacity, co-existing interstitial lung disease, or of young age, for patients with these conditions all face an up-to-15-fold increased risk of a second primary lung cancer following initial treatment.^[8]^ Some modalities such as Cyberknife® and Synchrony® technology can perform real-time tumor tracking with internal or external tracking, and can be used for SABR. MR-Linac provides sagittal MRI with good resolution without additional radiation dose and fiducial markers, and its automatic beam-gating technology can treat patients with small PTV even under free breathing conditions if the patient cannot hold his/her breath, which benefits both medical staff and patient.

MR-Linac is a newly developed modality that is ideal for lung SABR because of the following features: Superior soft tissue visualization compared with CT; Daily on-table adaptive replanning; Real-time continuous intrafraction assessment of internal structures; and Automatic beam-gating based on target position.^[9]^ Daily on-table adaptive replanning enables the physician to recontour the GTV and PTV daily for precision treatment. There are more and more studies referring to lung SABR with MR-Linac. A previous study has shown online adaptive MR-guided lung SABR can provide better target conformality and homogeneity and OAR sparing compared with non-adaptive SABR in selected cases.^[10]^ Real-time continuous intrafraction assessment of internal structures with automatic beam-gating based on the target position benefits lung SABR even more. Since no internal target volume (ITV) is needed by this technology, smaller PTV is created,^[8]^ and thus lesser volumes of normal lung parenchyma is irradiated. This advantage can be maximized in SABR for lung cancer because the fractional doses of lung SABR are much larger than those of conventional fractionated radiation therapy.^[11]^ Studies have shown that SABR for lung tumors with MR-Linac results in low rates of high-grade toxicity and encourages early local control, even in patients with high-risk features and central tumors.^[12]^

One of the most impressive features of the presented case is that the 2 lesions were treated at the same time with a single treatment plan, thereby shortening treatment time and avoiding risk of dose overlap compared with using 2 isocenters. Setup error can also be avoided with 1 isocenter during treatment. Patient can then receive the treatment safely and comfortably compared. Even though the 2 lesions were not so close to each other, the treatment was completed successfully with the help of real-time continuous intra-fractional MRI. There have been some studies using volumetric-modulated arc therapy (VMAT) with multi-isocenter for SABR to multiple lung tumors showing acceptable toxicities,^[13]^ which gave us confidence to perform SABR for this patient. Video-assisted MR-guidance by MR-Linac provides an attractive solution for the issue of respiratory motion since no ITV is needed, allowing for high precision and reproducibility of delivery.^[8]^ A dosimetry comparison study showed the average PTV volume of the MRI-based-intensity modulated radiation was approximately 4-fold smaller than that of commonly used VMAT, which has lower doses to bronchi, rib, ipsilateral lung and whole body compared to VMAT for lung SABR, especially for tumors located in the lower lobe as lower-lobe tumors appear to have greatest motion by breathing.^[11]^ The patient presented here had previously undergone a left lower lobectomy. The tumors presented here were located in the lower zone of the upper lobe, so proper target motion detection and management might have been crucial for reducing uncertainty and radiation field in our case. In summary, our patient was suitable for MR-guided SABR.

Some challenges in using MR-Linac for lung SABR remain. The resolution of MRI by MR-Linac is impaired due to the weaker magnetic field and might be affected by respiratory and cardiac motion. Secondly the MRIdian system currently provides only 1 MR sequence (True FISP), which provides a fast temporal resolution and allows for real-time monitoring of respiratory motion.^[12]^ This may be a disadvantage for most tumors since no other functional image can be used for assistance, but fortunately this is not the case for lung lesions. Thirdly, on-table real-time imaging for gated delivery is only presented in the sagittal view, so the lateral movement of the target cannot be detected.

Some other limitations for this study should be mentioned. Firstly, biopsies of the tumors were not performed after multidisciplinary discussion, which also followed the suggestion of guidelines for SABR to medically inoperable NSCLC.^[14]^ Secondly, we only tracked the larger tumor with real-time continuous intrafraction during treatment as a proper sagittal view to see both tumors clearly could not be realized. Fortunately, it was found that the 2 lesions moved consistently under free breathing by sagittal cine MRI in this case; however, simultaneous irradiation should be used carefully in highly selected cases to avoid missing the target. Thirdly, the lesions of our case might be too small for detection by PET, and the post-RT CT images still showed some pneumonitis, which might interfere with the interpretation of treatment response. After careful consideration, the tumors were determined to have had good response to SABR by both CT and PET images.

We present a patient receiving simultaneous SABR to multiple lung tumors with 1 single plan using MR-Linac. As far as we know, few cases with multiple lung tumors treated with SABR simultaneously have been reported,^[15]^ and there are a limited number of cases treated with single isocenter^[16]^ and MR-Linac.^[17]^ In our knowledge, the current manuscript is the first report of ViewRay MRIdian Linac used for 2 lung tumors. We believe that the present case provides experience and information using SABR for multiple lung tumors with single isocenter by MR-Linac without the need for ITV. With good response, limited adverse effect and shorter treatment time, simultaneous SABR to multiple lung tumors using MR-Linac could be an appropriate treatment for surgically inoperable patients.

## Author contributions

**Conceptualization:** Chih-Jen Huang.

**Data curation:** Frank Chen, Yuan-Kai Cheng, Chen-Han Chiang, Tzu-Ying Lu, Chih-Jen Huang.

**Investigation:** Frank Chen.

**Methodology:** Frank Chen.

**Resources:** Chen-Han Chiang, Tzu-Ying Lu.

**Validation:** Yuan-Kai Cheng, Chen-Han Chiang, Tzu-Ying Lu, Chih-Jen Huang.

**Writing – original draft:** Frank Chen.

**Writing – review & editing:** Chih-Jen Huang.
